# Maternal characteristics and infant and child mortality: A cohort study in England linking Census and health data

**DOI:** 10.23889/ijpds.v9i5.2690

**Published:** 2024-09-18

**Authors:** Isobel Ward, Sarah Barrat, Charlotte Standeven, Cameron Razieh, Emry John, Daniel Ayoubkhani, Vahe Nafilyan

**Affiliations:** 1 Office for National Statistics

Child mortality is a common measure of the overall health of society. Maternal characteristics, such as ethnicity and socioeconomic status are known to contribute to inequalities in health and mortality in babies and children. Overall, in the UK babies born to non-white mothers have a higher risk of mortality, and there is also a strong association between deprivation and risk of death. However, population data is currently lacking for England and Wales disentangling the various socioeconomic risk factors which are known to contribute to increased risk. We utilise population level data to assess the association between socioeconomic status, ethnicity, and child mortality.

Our cohort consisted of all live singleton births in England and Wales between 2011 and 2016. We linked birth notifications to mothers’ 2011 Census records using NHS number, which provides person-level sociodemographic information. Death registration data was linked to identify all-cause and cause-specific deaths in babies. Babies were followed from date-of-birth for up to 10-years, or date-of-death. We report cumulative incidence of all-cause mortality for a 10-year follow-up for neonatal, infant and child deaths based on maternal socioeconomic and ethnic groups. Cox proportional hazard models are used to estimate hazard ratios for neonatal, infant and child deaths, with both minimally adjusted (maternal age and baby sex) and fully-adjusted models (accounting for other maternal, household and birth characteristics).

This presentation will give an overview of our unique linked data sources, the statistical techniques employed, the latest available analytical results, and the emerging implications of the research.

